# Where Is the Vagina? A Rectal Stricture after a Presumed Cloacal Repair Turns Out to be the Mobilized Vagina and a Missed High Rectovaginal Fistula

**DOI:** 10.1055/s-0042-1755538

**Published:** 2022-10-10

**Authors:** Shimon E. Jacobs, Laura Tiusaba, Elizaveta Bokova, Tamador Al-Shamaileh, Teresa L. Russell, Briony K. Varda, Christina Feng, Andrea T. Badillo, Marc A. Levitt

**Affiliations:** 1 Department of Surgery, Division of Colorectal and Pelvic Reconstruction, Children's National Hospital, Washington, District of Columbia, United States; 2 Department of General Surgery, Division of Pediatric Surgery, King Hussain Cancer Center, Amman, Jordan; 3 Department of Urology, Children's National Hospital, Washington, District of Columbia, United States

**Keywords:** anorectal malformation, cloaca, rectovaginal fistula, cloacagram, imperforate anus

## Abstract

We present a case of a rare complication in a 10-month-old female referred to our institution for an anal stricture after primary cloacal repair as an infant. Multimodal imaging, careful physical exam, and endoscopic evaluation revealed her vagina had been pulled through to the location of her anal sphincter muscle complex. We describe the correction of this problem, including identification of her rectum.

## Introduction

Anorectal malformations (ARMs) can have a wide spectrum of anatomic complexity, and clear preoperative imaging is key to planning and executing an anatomic reconstruction. Common postoperative complications of ARM repair include rectal prolapse, remnant of the original fistula, anal or rectal stricture, wound dehiscence, and recurrent fistula. 1 A cloacal malformation is characterized by a single perineal orifice in which the urologic, gynecologic, and gastrointestinal tracts meet. It represents one of the most complex congenital anomalies of the pelvic organs in females, with an incidence of 1 in 50,000 births. 2 3 4 In 30 to 70% of nonsyndromic patients with ARMs, a combination of congenital anomalies of multiple systems known as the VACTERL (V—vertebral, A—anorectal, C—cardiac, TE—tracheoesophageal, R—renal, L—limb) association is present. The diagnosis of VACTERL association requires that three or more of these conditions are present. 5 6 7

## Case Report

A 10-month-old female was referred to our center for an anal stricture. She was diagnosed as an infant with a short channel cloaca. After initial colostomy and mucous fistula creation, she underwent a posterior sagittal anorectoplasty at an academic children's medical institution, with mobilization of what was thought to be the rectum. She reportedly did not require any surgical correction of the genitourinary sinus component of her cloaca.

On our exam, the urethral meatus was hypospadic, 0.5 cm inferior to the convergence of the corpora. Cystoscopy showed a normal urethra and bladder, but we could not identify the vagina. The perineum had an opening within the muscle sphincter complex, previously described as the anus that was severely strictured for a length of 1.5 cm, accommodating only a size 4 Hegar dilator.

**Fig. 1:**
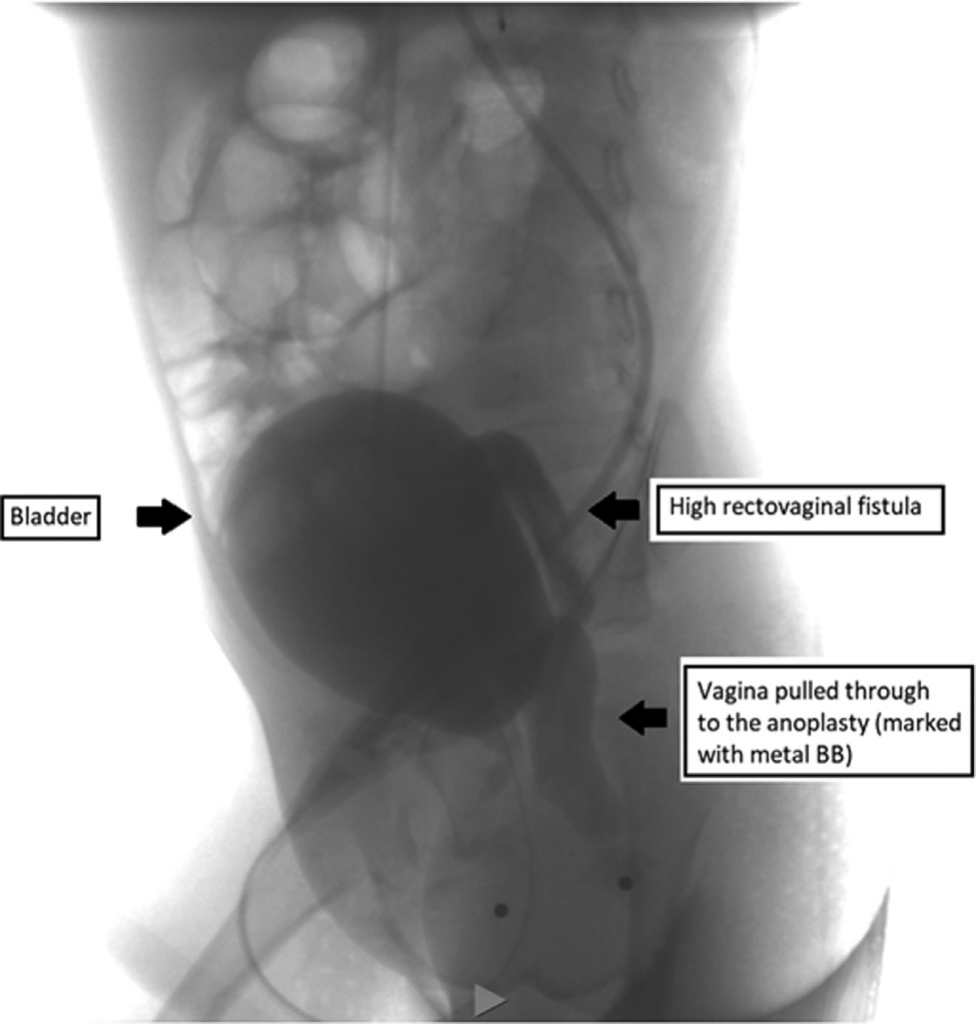
A distal colostogram with structures labeled. The two dots represent the urethral orifice anteriorly and the anoplasty location posteriorly.

**Fig. 2:**
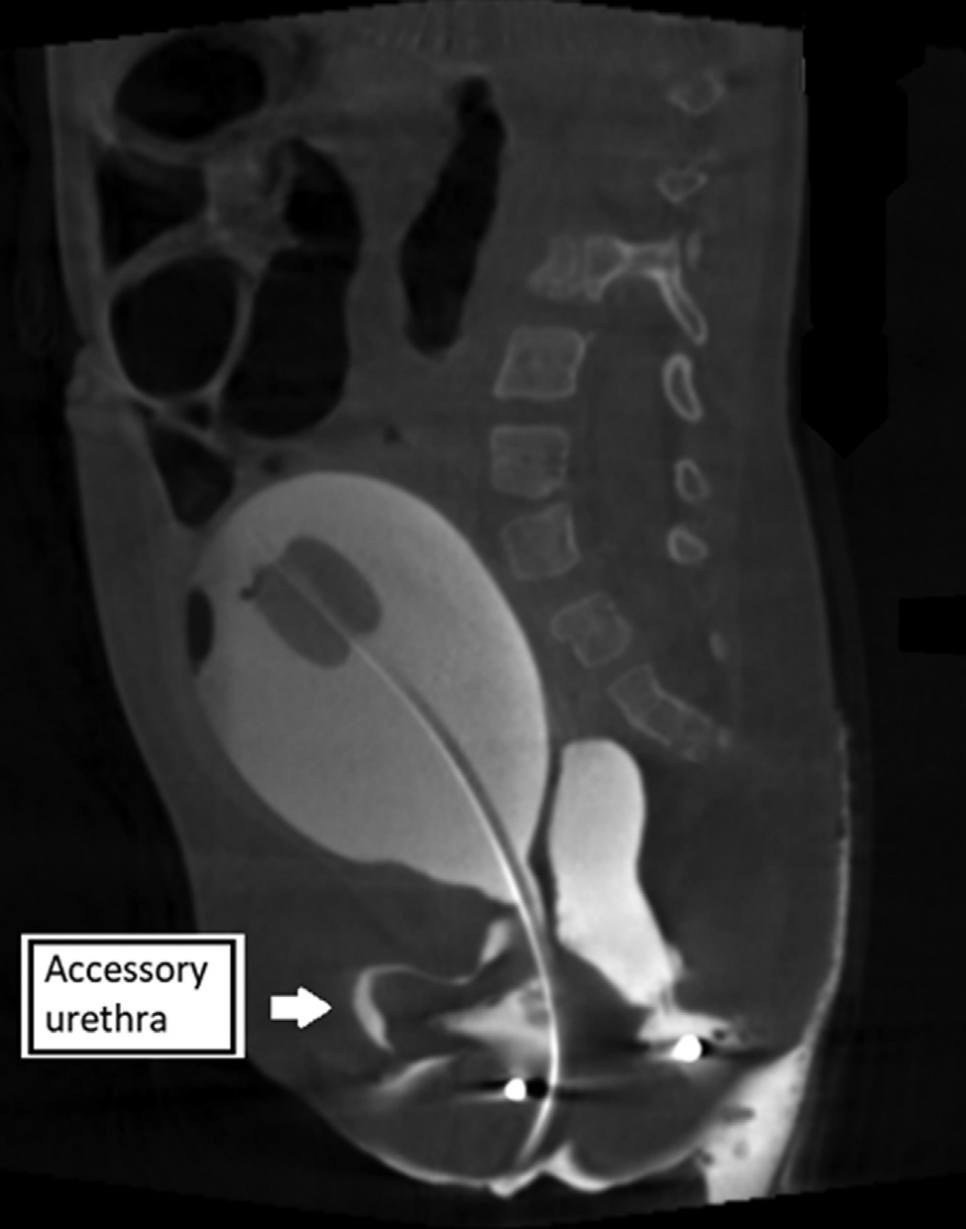
Three-dimensional cloacagram, which is rotatable, showing more detail and noting an accessory urethra anterior to the bladder, labeled here.

A distal colostogram ( Fig. 1 ) showed that the vagina had been mistaken for the rectum and had been pulled through as the “anoplasty.” The original rectum ended in a high rectovaginal fistula above the pubococcygeal line, which had not previously been recognized or repaired. Three-dimensional imaging confirmed these findings and showed an accessory anterior urethra ( Fig. 2 ). Additional urologic anomalies included a malrotated left kidney and mild bilateral hydronephrosis. Her sacral X-rays demonstrated three sacral elements with fusion of the lower elements and a lateral sacral ratio of 0.72. A spinal magnetic resonance imaging was obtained showing her conus medullaris terminating at the upper end of L3, and the neurosurgeon's review determined the cord was not tethered.

Based on our preoperative studies, we started the surgical reconstruction with a laparoscopic mobilization of the rectum and dissected out the rectovaginal fistula but left it attached to the posterior vagina. Inspection of the pelvis revealed an atretic hemiuterus on the left side, which was resected with the Fallopian tube, while preserving the left ovary. The right side had normal anatomy. After sufficient length on the rectum was obtained, the patient was then positioned prone. Via a posterior sagittal incision, a full-thickness mobilization of the native vagina around the “anoplasty” was performed. The vagina, once mobilized, was then placed at the introitus abutting the posterior urethra and urethral meatus. The rectum was detached from the back of the vagina, mobilized, and brought down into the anal muscle complex to create the anoplasty. The perineal body was then reconstructed. The orthotopic urethral meatus was visible, allowed for easy catheterization, and did not require any mobilization. The accessory urethra appeared blind ending, as it approached the glans clitoris. This will be observed with consideration of excision if clinically symptomatic.

## Discussion

Evaluation of an ARM patient who is not doing well postrepair starts with obtaining a detailed history including symptoms, current and past medical therapies, and surgical interventions. 8 This patient was referred for anal stricture, which occurs in 20 to 30% of ARMs. 9 A review of 95 redo operations in cloaca by this paper's senior author showed that the most common indication for revision was a persistent urogenital sinus in which the rectal component only was repaired (46), followed by a rectal stricture or atresia (45), acquired vaginal stricture or atresia (45) mislocated rectum (36), urethrovaginal fistula (16), rectal prolapse (12), urethral atresia or stricture (7), and rectovaginal fistula (5), with most patients having greater than one indication. 10 The first challenge in reducing complications of cloacal repair is to determine the complexity of the malformation before embarking on the repair. To optimize the functional result, a key part of the evaluation is to confirm that the anatomy has been correctly reconstructed.

At our institution, endoscopy is routinely performed for patients with complex malformations to understand the anatomy of the bladder, ureters, urethra, vagina(s), and common channel (in the case of a cloaca), as well as to visualize the location and height of the distal rectum. Anomalies of the genital tract such as a vaginal septum, bicornuate uterus, or uterine didelphys may be diagnosed.

Three-dimensional reconstructed fluoroscopy or cloacagram is performed to obtain the lengths of the common channel and urethra and to assess spatial relations of the rectal fistula and other structures relative to the pelvis. 11 In the initial surgical repair of this patient, it is likely that the first structure dissected during the posterior sagittal incision was believed to have been the rectum, located at the vestibule, instead of correctly identifying that structure as the vagina. Our imaging showed the location of the original rectum to be above the pubococcygeal line, making the posterior sagittal approach of the rectal dissection exceedingly difficult, and an initial laparoscopic mobilization preferred. The distal rectum and fistula may not have been clearly delineated on the original preoperative colostogram, contributing to this misidentification. A true rectovaginal fistula is exceedingly rare and a cloacal malformation should be suspected in a female patient with imperforate anus without a clear vestibular or perineal fistula, and without a normal urethral orifice. Preoperative planning with a multidisciplinary approach, including endoscopy and multimodal imaging, is vital to achieve the successful, definitive anatomic repair of ARMs.

Two months after her reoperation, this patient's colostomy was closed. At her follow-up appointment 9 months postoperatively, she was experiencing mild constipation, which was well managed with stimulant and mechanical laxatives.

## Conclusion

This case demonstrates a rare complication that occurred during what was thought to be a cloacal reconstruction in which the vagina was pulled through as the anoplasty. Multimodal imaging allowed us to delineate the anatomy and plan a successful reconstructive strategy to ensure a repair that maximized the patient's potential for fecal continence as well as gynecologic function. This case is an important reminder of the need for correct and comprehensive preoperative imaging and anatomical evaluation prior to performing a reconstruction for an ARM.

## Notes

New Insights and the Importance for the Pediatric Surgeon Multimodal imaging allowed for delineation of the postoperative anatomy of a cloacal repair presenting with a complication, and formulation of a plan for a reconstructive strategy to ensure a repair that maximized the patient's potential for fecal continence as well as gynecologic function. This case is an important reminder of the need for correct and comprehensive preoperative imaging and anatomical evaluation prior to performing a reconstruction for an anorectal malformation.
